# Innovative Strategies to Improve the Clinical Application of NK Cell-Based Immunotherapy

**DOI:** 10.3389/fimmu.2022.859177

**Published:** 2022-03-25

**Authors:** Mubin Tarannum, Rizwan Romee, Roman M. Shapiro

**Affiliations:** Division of Stem Cell Transplant and Cellular Therapy, Dana Farber Cancer Institute, Harvard Medical School, Boston, MA, United States

**Keywords:** natural Killer (NK) cell, immunotherapy, *in vitro* expansion, tumor infiltrating cell, NK cell exhaustion

## Abstract

Natural killer cells constitute a part of the innate immune system that mediates an effective immune response towards virus-infected and malignant cells. In recent years, research has focused on exploring and advancing NK cells as an active immunotherapy platform. Despite major advances, there are several key challenges that need to be addressed for the effective translation of NK cell research to clinical applications. This review highlights some of these challenges and the innovative strategies being developed to overcome them, including *in vitro* expansion, *in vivo* persistence, infiltration to the tumor site, and prevention of exhaustion.

## Introduction

Natural killer (NK) cell-based therapies are a promising modality for the treatment of liquid and solid tumors. With their intrinsic ability to mediate both cytotoxicity as well as cytokine secretion that helps mobilize other immune effector cells, NK cells are a powerful tool in the armamentarium of cancer immunotherapy (1, 2). While the initial recognition of their anti-tumor efficacy came from studies of allogeneic stem cell transplantation, numerous other studies have demonstrated their cytotoxic potential against tumor targets even in non-transplant settings (3, 4). In recent years, great progress has been made in the *ex vivo* activation and expansion of NK cells for clinical applications, as well as their manipulation with exogenous cytokines and genetic methods to further enhance cancer targeting (5–8). While this progress has led to the translation of several NK cell-based immunotherapies into early phase clinical trials, ongoing studies aim to overcome persistent challenges with this therapy. The purpose of this review is to provide an overview of the strategies being developed to address these challenges, including optimization of NK cell expansion, persistence, trafficking, and anti-tumor activity in the tumor microenvironment (TME).

## Strategies to Generate and Expand NK Cells

**Figure 1:**
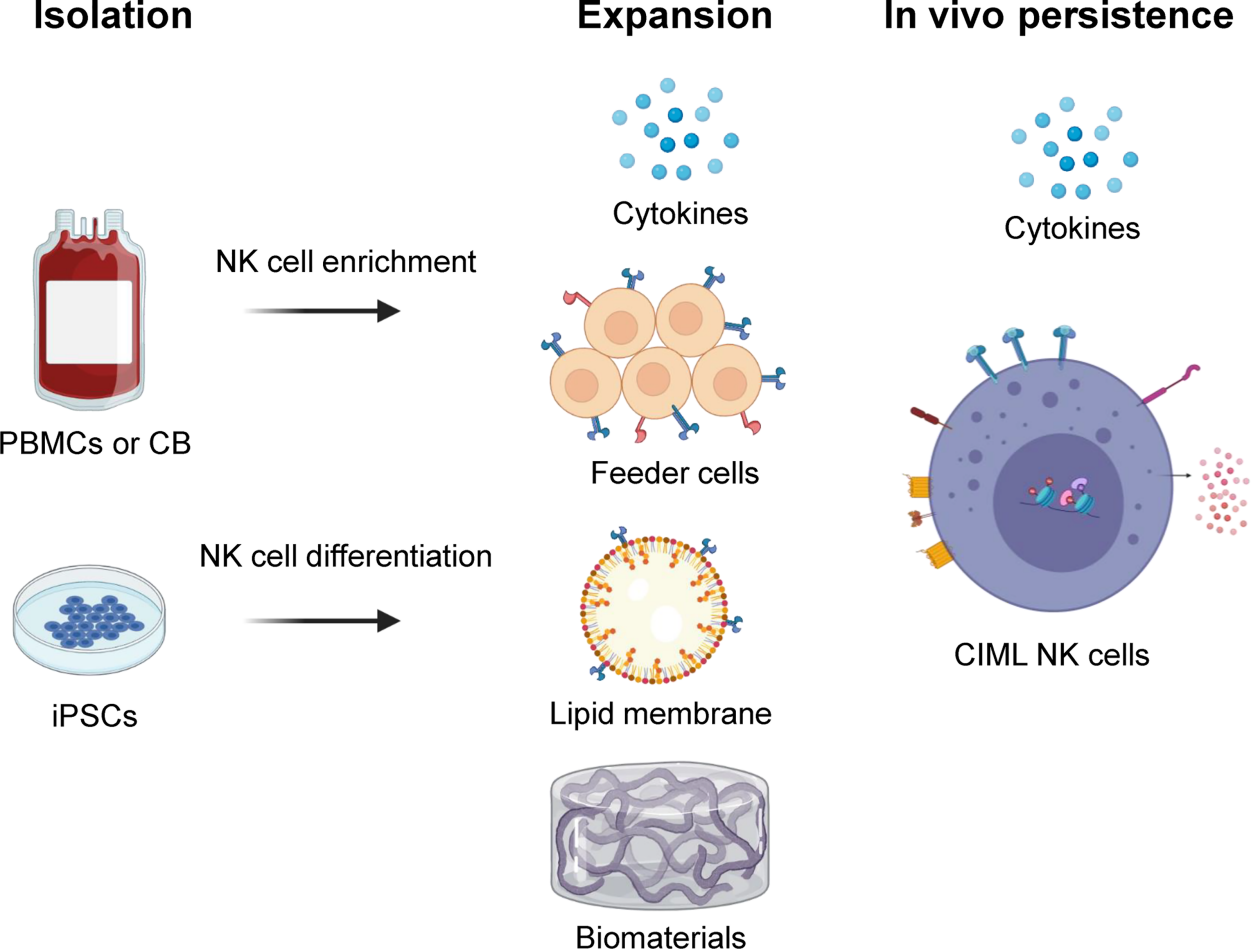
Overview of NK cell isolation, expansion, and *in vivo* persistence. NK cells can be isolated and/or generated from peripheral blood (PB), cord blood (CB), or human induced pluripotent stem cell (iPSC) sources. They are expanded *ex vivo* using various strategies including cytokine combinations, feeder cells, lipid membranes or biomaterials that carry surface markers to provide biochemical cues. *In vivo* persistence of NK cells can be accomplished using cytokines like IL-15 and/or cytokine-induced memory-like (CIML) NK cells that have shown long term persistence in an immune compatible environment. Illustration created using BioRender.com.

The major goal for NK cell expansion strategies is to generate large numbers of these cells for clinical applications that retain high anti-tumor activity. With substantial NK cells, it is possible to achieve higher desirable NK effector to target ratios, perform multiple infusions, and potentially allow for the development of off-the-shelf NK cell products. There are multiple NK cell expansion strategies and sources for generating clinical grade NK cell products including peripheral blood (PB), cord blood (CB), bone marrow (BM), and human induced pluripotent stem cells (iPSC) (**Figure 1**) (9). NK cells can be expanded *ex vivo* before infusion into patients, or *in vivo* after adoptive transfer with cytokine stimulation. They are typically isolated by CD3^+^ cell depletion followed by positive selection of CD56^+^ cells, or in some protocols by single step depletion of CD3^+^ and CD19^+^ cells using magnetic beads (CliniMACS) (10). Another approach includes differentiation of functional NK cells from enriched CD34^+^ progenitors present in CB and BM (**Table 1**) (11, 29).

**Table 1:** Advantages and disadvantages of different methods of NK cell expansion for adoptive transfer studies.

Source of NK cells	Activation or stimulation	Advantages	Disadvantages			Refs
***Ex vivo* Expanded**						
Umbilical cord		Higher percentage of NK cells than other sources	Relatively low number of NK cells		(11, 12)	
blood		Contains NK progenitors that can differentiate into NK cells, and that are absent from	Potentially lower cytotoxicity that PB derived NK cells			
		Have higher expression of bone marrow homing receptors				
iPSC		Can be generated in an off-the-shelf manner	Requires specialized expertise to generate NK cells from iPSC and to maintain clinical grade iPSC cell lines		(13, 14)	
		iPSCs can be propagated indefinitely and can generate NK cells for multiple treatments	Relatively low level in the peripheral blood after infusion	
		Generate purer NK cell populations with known KIR haplotypes		
Peripheral blood (CD3 depletion, CD56 enrichment) CD3/CD19 depleted	IL-2 stimulation	Enhanced viability after cryopreservation, enhanced cytotoxicity	Short-term treatment (12-16 hours) does not result in sufficient NK cell expansion on its own		(15, 16)	
	IL-15 stimulation	Enhanced NK cell cytotoxicity	Can result in functional exhaustion of NK cells if stimulation is too long			
	IL-2 + IL-21 stimulation	IL-21 enhances NK cell cytotoxicity, cytokine secretion, and ADCC	IL-21 may trigger NK cell apoptosis			
	IL-12 + IL-15 + IL-18 stimulation	Generates memory-like NK cells				
		Increases expression of CD25 on NK cells, making them more amenable to IL-2 mediated expansion		
	IL-15 + IL-21 stimulation	Method can be combined with CD34 selection to concurrently purify stem cells for infusion	Contain other contaminating cells such as monocytes and dendritic cells		(17, 18)	
CD34+ selection followed by differentiation to NK cells		Generates high purity of NK cells	Greater variability and poorer yields of differentiated NK cells than from umbilical cord blood		(19)	
		Can generate more CD34+ cells than umbilical cord blood		
**Feeder cell lines**						
	Irradiated autologous PBMCs		Limited availability as the feeder cells must be obtained from a pretreated patient	(7, 8, 14, 20–23)		
			Generally poorer expansion than from allogeneic donor PBMC				
	Irradiated K562 with membrane bound IL-15/4-1BB/IL-2	Enhanced NK cell cytotoxicity	Increased incidence of aGVHD after infusion into patients in one trial				
		Efficient ADCC				
	Irradiated K562 with membrane bound 4-1BB/IL-21	Increased telomere length	Allogeneic feeder cell lines must pass stringent quality control measures in order to be acceptable for infusion into patients			
		Enhanced persistence				
	Plasma membrane particles of K562 with membrane bound IL-21/4-1BB/IL-2	Lower risk of transmission of feeder culture contaminant to patient	NK cells expanded with plasma membrane particles tended to have a lower expression of the bone marrow homing molecule CD62L compared to feeder culture cell line expansion			
		Similar cytotoxicity profile to those expanded with feeder culture				
	EBV transformed lymphoblastoid cells	Enhanced NK cell cytotoxicity				
		Increased NK cell cytokine secretion				
	Priming cell lines	Primed NK cells are KIR independent and do not require cytokine stimulation		(15, 24)		
	CTV-1 leukemia cell line					
**Biomaterials**						
	Hyaluronic acid-based biodegradable polymeric scaffold					(25)
***In vivo* Expanded**						
CIML NK cells	expanded with low dose IL-2	Massive *in vivo* expansion	Specialized processing facility with high manhours required			(21, 26, 27)
		Enhanced cytotoxicity				
NK	IL-2	Expansion of NK cells	May also expand regulatory T-cell populations			(28)
			Associated with capillary leak syndrome, constitutional symptoms and cardiac toxicity		
NK	IL-15	Expansion and persistence of NK cells	The subcutaneous form is associated with neurologic toxicity and cytokine release syndrome			(28)

Although good manufacturing practice guidelines have been defined for NK cell products for clinical applications, the source of NK cells does result in some important differences with respect to NK cell numbers and phenotype (**Table 1**) (15, 30). It is currently not clear if these differences impact clinical outcomes. For example, while CB has a higher proportion of NK cells than PB, the total NK numbers that can be enriched from CB are significantly lower. Similarly the proportion of less mature CD56^bright^ cells is higher in CB as compared to PB (11). NK cells can also be generated *via* differentiation of CB-derived CD34+ cells. This process yields a greater expression of CXCR4 on the differentiated cells that mediates bone marrow homing (31). Killer Ig-like receptor (KIR) expression is lower when CD34+ progenitors are differentiated into NK cells that may impact their tumor-targeting ability (20). Furthermore, while CB-derived NK cells seem to have comparable cytokine secretion in response to the *in vitro* target cell stimulation, they exhibit lower cytotoxicity than PB-derived NK cells (11).

Differentiation and expansion of NK cells from iPSC is another promising approach that has seen significant advancement for clinical application in recent years (13, 14). While the generation of a sufficiently large number of NK cells from iPSC requires a longer time than PB, it is particularly attractive in terms of establishing well characterized master banks, performing multiple gene edits (including CAR arming and CRISPR) and generating functional immune effector cells in large numbers for off-the-shelf use. iPSC-derived NK cells exhibit comparable cytokine-secreting and cytotoxic capacity to PB-derived sources. They are able to infiltrate tumor spheroids *in vitro*, interact with other effector T-cells in mouse models and are amenable to gene editing with CRISPR technology (13, 32). A phase 1 clinical trial evaluating the feasibility and efficacy of iPSC-derived NK cells is currently ongoing (NCT03841110).

### Cytokine-Based *Ex Vivo* Activation and Expansion of NK Cells

Traditionally IL-2 and more recently IL-15 have been added to culture media to support activation and expansion of purified NK cells (21, 33). IL-2 promotes NK cell expansion and survival as both CD56^dim^ and CD56^bright^ subpopulations express the high affinity IL-2 receptor (34). However, the effect of IL-2 is not specific to NK cells and may also expand regulatory T-cells (Tregs) (35). IL-15, while sharing similar signaling functions with IL-2, is selective in activating NK cells and CD8^+^ T cells, and is therefore preferable for NK cell activation and expansion efforts (34).

Other *ex vivo* expansion methods have been developed, including the use of feeder cells and biomaterials (**Figure 1**). The use of membrane bound IL-15 and 4-1BB on K562 feeder cells have been shown to result in significant expansion of NK cells (36–38). A similar method incorporating membrane-bound IL-21 instead of IL-15 on K562 feeder cells (K562-mbIL21) has the advantage of preserving the telomere length of expanded NK cells, thereby resulting in the infusion of cells with a more sustained proliferative potential (7, 39, 40). A phase 1 trial of adoptively transferred K562-mbIL21 expanded NK cells infused peri-transplant has shown such an approach to be safe and promising (41, 42). Another study evaluated a similar approach of infusing NK cells expanded with cell-free liposomal plasma membrane particles containing mbIL21 and 4-1BB (43). Other feeder cell types have been developed including the Epstein-Barr lymphoblastoid cell line and human B-lymphoblastoid cell line 721.221, with variable effects on the expansion of NK cells while preventing the concurrent expansion of T-cells (44, 45).

### Biomaterials for Activation and Expansion of NK Cells

Along with targeted drug delivery, unique properties of biomaterials can be exploited for cancer immunotherapy, especially to support the activation and expansion of immune cells. Advances in the engineering of biomaterials have helped to study the interaction of immune cells and precise control of their immunomodulatory functions (46). Various biomaterials including lipid bilayers and nano/micropatterns have been used to study the fundamental mechanisms of immune cell-target interactions in NK cells, providing new insights on the interaction of putative NK ligands with specific activating or inhibitory receptors (22, 47–49). In addition, these materials can be engineered to provide synthetic molecular, physical, and biochemical cues to support the growth of NK cells. Specifically, the surface of the scaffolds generated from these materials can be modified/decorated with specific receptors supporting NK cell expansion or loaded with chemokines/cytokines for their sustained release. Such material designs can provide both co-stimulatory signals and cytokine support for precise immunomodulation, aimed at providing cells with a synthetic environment similar to their natural biological environment (50).

Various materials have already been designed and investigated for T cell expansion including polymer beads, alginate scaffold, mesoporous silica rods (MSR), polyisocyanopeptide scaffold, and hydrogels (51). Although there are limited studies of such scaffold based systems for NK cells, some studies used CD16-functionalized graphene oxide nanoclusters for the *in vitro* activation of NK cells (52). In a recent study by Ahn et al, NK cells were expanded using a hyaluronic acid-based biodegradable polymeric scaffold with macro-porous 3D structure (25). The macro-porous architecture supported the cluster formation of loaded NK cells resulting in their proliferation and persistence (25). For the clinical translation of biomaterials, key parameters need to be considered including material composition, biodegradability and the toxicity of chemicals post-degradation, and intrinsic immunogenicity. In addition, the physiochemical properties like surface area and topology, porosity, and ease of functionalization of the material are vital for their application (51, 53).

## *In vivo* Persistence of NK Cells After Infusion

The efficacy of NK cell therapy depends in part on the ability of the infused cells to persist *in vivo* after adoptive transfer (54). While NK cells could be expanded in large numbers *ex vivo*, following infusion these NK cells exhibit limited *in vivo* persistence and expansion (55). Efforts to expand and activate NK cells after their adoptive transfer have typically relied on the cytokines IL-2 or IL-15, the latter being somewhat favored because of its greater selectivity for NK cells (56). A super-agonist complex (ALT-803) of an IL-15 mutein bound to the sushi domain of the IL-15 receptor alpha (IL-15Rα) and fused to the immunoglobulin G1 Fc region has been generated that in addition to having a longer half-life and improved functionality over the individual cytokine (57), resulted in marked expansion and activation of NK cells in a phase I clinical trial (58).

The expansion of NK cells using feeder cell lines such as K562, Epstein-Barr lymphoblastoid cell line, and human B-lymphoblastoid cell line 721.221 that either express membrane-bound cytokines or in co-culture with exogenously administered cytokines such as IL-21 are methods developed for the generation of activated NK cells with a potential for long-term persistence *in vivo* (39, 41, 43–45, 59). In addition, genetic manipulation of NK cells *via* viral and non-viral techniques for autocrine expression of cytokines (IL-15 or IL-2) has been shown to increase the cells’ persistence and expansion post infusion (60).

Differentiation into a memory-like phenotype is another novel approach that results in normally short-lived NK cells persisting for much longer periods with significantly increased *in vivo* expansion and anti-tumor responses (6, 21, 61–63). Cytokine induced memory-like (CIML) NK cells generated after brief activation with cytokines IL-12, IL-15, and IL-18 exhibit this unique memory-like phenotype (**Figure 1**). In preclinical and recent clinical settings, CIML NK cells have demonstrated massive expansion, prolonged persistence, and reduced threshold for activation in response to tumor targets (64).

Ongoing clinical studies have shown that the prolonged persistence of the adoptively transferred CIML NK cells is associated with the attainment of clinical responses (6, 21, 61–63). Upon their adoptive transfer into an immune compatible environment in the context of an allogeneic HCT in a phase 1 clinical trial, the infusion of CIML NK cells resulted in a massive expansion of the NK cell compartment that persisted for up to 6 months (26). The use of the CIML NK platform for the prevention of post-allogeneic HCT relapse of myeloid disease is concurrently being explored (65).

## Strategies to Enhance NK Cell Infiltration and Targeting in Solid Tumors

In contrast to hematological cancers, solid tumors are characterized by a dense, highly heterogeneous, stroma-rich TME within which crosstalk between the cancer cells and their microenvironment helps drive tumor progression, invasion, and metastasis (66, 67). The TME is composed of cellular and acellular components which often hinder the delivery of small molecules and infiltration of infused adoptive cells (68, 69). NK cell infiltration in the tumor is hindered by lack of chemotactic signaling and activation, as well as by the physical barriers in the TME due to hypo-vasculature and dense extracellular matrix (ECM) deposition (69). In addition, myeloid-derived suppressor cells (MDSCs), tumor associated macrophages (TAMs) and Tregs are often enriched within the advanced tumor sites, and play a key role in inhibiting immune effector cells such as NK and CD8^+^ T cells (66). The tumor-stromal interaction results in the release of immunosuppressive molecules including IL-10 and TGFβ, the downregulation of antigens targeted by immune effector cells and the upregulation of checkpoint blockade proteins. All these molecules shape the TME to be highly immune-suppressive, thereby hampering effective antitumor immune responses (66). To mediate optimal anti-tumor responses in the solid tumor setting, circulating NK cells need to exit the bloodstream, traffic to tumor sites that include distant metastatic sites, penetrate through the dense ECM, and finally interact with and respond to the tumor cells. Previous reports have shown that only a minority of adoptively-transferred lymphocytes traffic to the tumor sites (70, 71).Various strategies have been investigated to potentially overcome this major barrier, including utilizing chemokine networks for efficient NK cell infiltration into the TME, imparting features to NK cells to overcome the immunosuppressive TME, as well as altering the TME to create a favorable niche for the adoptively transferred immune cells (72).

### Key Chemokine Networks Involved in Trafficking of NK Cells to the Tumor Sites

**Figure 2:**
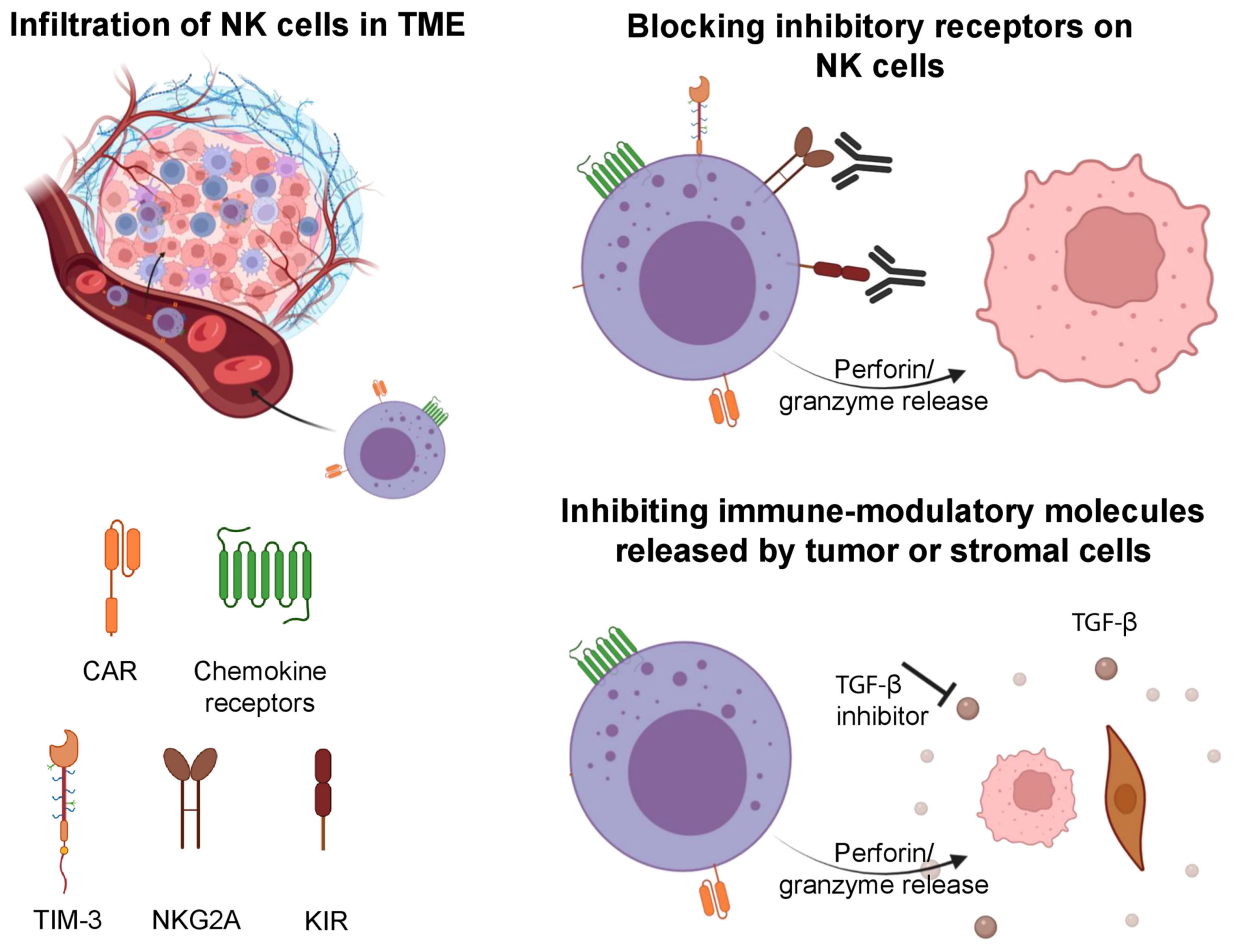
Strategies involved to improve the tumor infiltration of NK cells, overcome tumor escape and exhaustion. NK cells can be engineered to express CAR and specific chemokine receptors to increase their infiltration in solid tumors. Upon entry into the TME, NK cells can be impaired by its immune suppressive features. Blockade of inhibitory receptors (TIM-3, NKG2A, KIR) on NK cells can increase their antitumor cytotoxicity. Further, inhibition of immune-modulatory molecules (TGF-β) can prevent exhaustion in NK cells and maintain their cytotoxic features. Illustration created using BioRender.com.

Interaction between soluble chemokines and their receptors play an essential role in the migration of lymphocytes (73). The role of these receptors on NK cells, including CCR2, CCR5, and CXCR3 was described in a recent review (73). CCR2 has been shown to promote the recruitment of NK cells into lymph nodes infiltrated with melanoma (74). The expression of CCR5 on infiltrating immune cells was correlated with high levels of CCL5 in tumors, and was associated with improved overall survival (75). Alteration of the chemokine receptor profile on NK cells has been shown to drive their migration and infiltration into solid tumors (**Figure 2**). For example, autophagy inhibition in melanoma leading to PPA2-dependent upregulation of JNK phosphorylation led to increased expression of CCL5 on tumor cells, which in turn increased infiltration of CCR5 expressing NK cells into the TME (73). The latter was associated with improved outcomes in preclinical studies (76).

Based on the recent advances in our understanding of chemokine networks, NK cells have been genetically engineered with altered expression of chemokine receptors to modulate their homing into solid tumors or metastatic sites (64). Li et al. showed that transduction of primary NK cells with CCR5 resulted in their increased infiltration and subsequent anti-tumor activity in colorectal cancer cells-derived xenograft mice (77). On the other hand, NK cells expanded in the presence of K562 feeder cells engineered to express CCR7 resulted in the acquisition of CCR7 receptors by NK cells *via* trogocytosis (78). These CCR7^+^ NK cells, when adoptively transferred into athymic nude mice, exhibited enhanced homing into lymph nodes and increased cytotoxicity against tumor cells (78). In another study, the increased expression of CXCR1 in NK cells resulted in increased migration and infiltration into subcutaneous and intraperitoneal tissue in a model of ovarian cancer (79). Similarly, NK-92 and primary NK cells transduced to overexpress CXCR2 improved their migration and accumulation at sites of disease in models of renal cell carcinoma and lung cancer metastasis, respectively (80). Yang et al. demonstrated that more than one chemokine pathway is needed for the successful infiltration of NK cells in solid tumors (78). The authors showed that increased expression of CXCR4 and CCR7 on NK-92 cells significantly increased their migration in a colon cancer model (81). The level of chemokine receptor expression could also be induced by cytokine activation. For example, expansion of NK cells in the presence of IL-2 significantly increased CXCR3 expression resulting in increased migration towards CXCL10-producing tumor cells (82). In addition to cytokines, treatment of NK cells with TGFβ1 was reported to modulate the expression of chemokine receptors including CXCR3/CXCR4 which further increased their infiltration in tumors (83).

Another approach for enhancing NK cell infiltration into the tumor sites involves manipulation of the immune-suppressive TME to create a favorable niche for infused NK cells (73). A number of studies have demonstrated that intratumorally injected IFNγ induce expression of CXCL9, CXCL10, and CXCL11 in the TME, which in turn increases the local infiltration of NK cells (84). Park et al. developed a very interesting strategy wherein immunomodulatory microspheres, composed of biodegradable PLGA polymer, were engineered to release IFNγ into the TME (85). Intra-arterial transcatheter delivery of these microspheres resulted in local delivery of IFNγ and a significant increase in the NK cell infiltration as demonstrated in an orthotopic liver cancer model (85). Similarly, intratumoral injection of recombinant human IL-12 in patients with head and neck squamous cell carcinoma (HNSCC) showed increased NK cells and IFNγ expression in the tumors and lymph nodes compared to the control arm, which correlated to the overall survival (86).

### Arming NK Cells With Chimeric Antigen Receptors to Enhance Tumor Immunotherapy

Augmentation of NK cell targeting of tumors can be accomplished with a chimeric antigen receptor (CAR) (60, 87, 88). With recent improvements in the efficiency of retroviral transduction of NK cells and the engineering of CARs, this approach has become a reality. The promise of this approach was demonstrated with the use of CAR-transduced NK cells in advanced lymphoma patients (60). NK cells were purified from CB, expanded with IL-21 and 4-1BB expressing K562 feeder cells in the presence of IL-2, and subsequently infused into patients following lymphodepleting chemotherapy. Infusion of these cells was associated with clinical responses in 8/11 patients. Most responses to CAR-NK occurred within the first 1-2 months and attainment of negative minimal residual disease (MRD) complete remission appeared more likely at the higher infused cell doses. The presence of the CAR was detectable for several months after infusion, although the absolute number of persistent NK cells was not quantified in this study. As the maximum tolerated dose of infused CAR-NK cells was not reached in this trial and a higher effector-to-target ratio plays a role in NK cell efficacy, it is possible that a higher infused NK cell dose or improvement in their *in vivo* persistence may result in the attainment of more sustained MRD-negative CR (60).

Recently there have been efforts to use gene-edited iPSCs for differentiation into NK cell CARs (89, 90). iPSC-derived NK cells can more stably express CARs, and may serve as a better off-the-shelf option for the generation of CAR-NK than CB (13, 14, 20). Whether their more immature phenotype and generally lower KIR expression would be a barrier to efficacy in the context of a CAR remains to be determined. Furthermore, current active research aims to use CIML NK cells as a platform for CAR generated products, with their enhanced cytotoxicity and long-term persistence potentially augmented *via* the incorporation of a CAR (27, 64). Recent progress and improvements in CAR-NK cell technology have been reviewed in detail by our group and others (87, 88, 91). In addition to CAR, genetic engineering of NK cells has been useful to increase their antitumor efficiency as recently reviewed by our group and others (88, 92).

## Overcoming Mechanisms of Tumor Escape and Exhaustion

Early phase NK cell clinical trials have shown favorable safety profiles and promising responses using advances in NK cell engineering, including the differentiation into memory-like phenotype or arming with CARs. However, these studies have a common feature of an initial reduction of tumor burden followed by eventual reappearance of detectable disease in the majority of treated patients (21, 60, 93). These observations can be partially attributed to the tumor escape mechanism and exhaustion of adoptive NK cells (72).

The transformation of normal cells to malignant cells requires elaborate adaptations and oncogenic changes that allow a tumor to bypass detection and elimination by immune effector cells (94). Some of these adaptations involve downregulation or shedding of ligands that activate NK cells (e.g. MICA/B), release of immune-modulatory molecules from tumor cells or supporting stromal cells (e.g. TGFβ), and epigenetic changes (e.g. histone deacetylases, HDACs) (95, 96). Escape of tumors from NK cells must be overcome for NK cell-mediated therapies to become standalone treatments without the need for subsequent consolidation with other therapies. Current strategies to overcome tumor escape include but are not limited to the reversal of NK cell anergy, the combination of NK cell therapy with antibody-mediated therapy that can engage their ADCC function, and the development of off-the-shelf NK cells that may be transduced with CARs that can target escaping tumor cells (**Figure 2**) (72).

A lack of the KIR-HLA class I interaction (KIR-ligand mismatch) is associated with enhanced NK activity, and one strategy used by tumors to escape NK cell mediated effects involves the increased expression of HLA class I molecules on their surface (97). To overcome this, anti-KIR antibodies such as IPH2101 (previously known as 1-7F9) that bind the inhibitory KIR2DL1, -L2 and -L3 as well as the activating KIR2DS1 and -S2, and interefere with the receptors’ interaction with HLA-C have been developed (98). Lirilumab, a fully human IgG4 anti-KIR2D monoclonal antibody that recognizes the same epitope as IPH2101 and in a similar manner blocks the KIR interaction with HLA-C, has been well-tolerated in early phase clinical trials (99, 100) and has shown some clinical activity when combined with azacytidine in high risk myelodysplastic syndrome (MDS) (101). Similarly, the anti-NKG2A antibody monalizumab has been shown to increase the effector function of NK cells, and its combination with PD-1/PD-L1 blockade yielded evidence of clinical activity in early phase trials (102, 103). An alternative approach to blocking antibodies that targets the KIR-HLA class I interaction is to genetically engineer the NK cells to downregulate inhibitory signals including NKG2A and KIRs (104–107).

The antitumor response of NK cells can also be manipulated using monoclonal antibodies engaging activating receptors like CD16A, NKG2D, and NKp46 (108–110). Antibody dependent cellular cytotoxicity (ADCC) of the target cells coated with IgG molecules is mediated by NK cells through engagement of CD16 (Fc receptor) on NK cells (111). Based on a similar concept, novel NK cell engagers (NKCE), synthetic molecules composed of the fragments of monoclonal antibodies that simultaneously engage NK activating receptors and tumor antigens, have been designed (112, 113). Bispecific killer cell engages (BiKEs) containing ScFv fragments that target CD16 and a tumor antigen are one example of this, and have been used to target EpCAM on carcinomas (114), CD133 on cancer stem cells (115), and CD30 on lymphomas (116). AFM13 (CD16/CD30) is the first-in-class tetravalent bispecific molecule targeting CD30-positive lymphoma, and has shown promising antitumor responses in recent clinical trials when used as a monotherapy as well as in combination with allogenic NK cells (116, 117). This molecule belongs to the class of redirected optimized cell killing (ROCK) platform, which is equipped with high affinity towards specific epitopes on CD16, is independent of individuals’ CD16A allotype, not inhibited by serum IgG, and also prevents NK fratricide (118).

Similarly, trispecific killer engagers (TriKE) have been developed that consist of two scFv fragments connected by an IL-15 domain, enhancing NK cell-mediated anti-tumor efficacy by engaging CD16 on the NK cells and an antigen on the tumor cells. The GTB-3550 TriKE (CD16/IL-15/CD33), for example, has been shown to enhance NK cell proliferation and function, and is being evaluated in clinical trials (119). Other TriKE molecules targeting CD133 and B7-H3 have also shown promising efficacy in cell lines and mouse models (120, 121). The targeting of CD16 on NK cells by BiKEs and TriKEs may be limited by the known process of CD16 shedding (122). However, the combination of the NKCE with novel approaches that interfere with the shedding process are being investigated (113).

While engagement of CD16 has been one successful strategy of NKCE, other engagers have been designed to bridge the NKG2D activating receptor with tumor targets such as CS1 on multiple myeloma (MM) and HER2 on breast cancer cells (123, 124). Alternative NKCE designs, such as those bridging NKp30 and NKp46 with tumor antigens for example, have been reviewed recently (112). Trifunctional NKCE that co-engage two different NK activating receptors, such as CD16 and NKp46, with a tumor target antigen have also been developed, and have shown impressive *in vitro* activity and suppression of tumor growth in mouse models (110).

A plethora of immune-suppressive pathways in the TME combine to inhibit NK cell function. Exhausted or impaired NK cells in the TME exhibit downregulation of effector cytokines, decreased granulation, downregulation of NKG2D, a decrease in transcription factors like Eomesodermin and T-bet (125), and upregulation of inhibitory receptors such as PD-1, TIM-3, TIGIT and NKG2A (72). Expression of the latter markers on NK cells have been correlated with decreased NK cell functionality and importantly, various studies demonstrate that blockade of these receptors can increase NK cell cytotoxicity and function (72, 126).

TGF-β is one of the important cytokines that plays a critical role in the immune-suppressive TME. It limits NK cell function by inhibiting the T-bet transcription factor (SMAD3) and by targeting the mTOR pathway (127). In addition, TGF-β has been reported to contribute to the selective recruitment of non-cytotoxic CD56^bright^ NK cells by modulating the chemokine repertoire in tumors (127). To potentially overcome TGF-β mediated immune suppression, Otegbeye et al. combined *ex vivo* expanded NK cells with the TGF-β inhibitor LY2157299, resulting in a significant decrease in liver metastasis as observed in a colon cancer model (128). Another strategy by Burga et al. was to engineer a variant of the TGF-β receptor where the receptor domain was coupled with NK specific activating domains (129). The authors showed that these engineered NK cells could efficiently convert inhibitory TGF-β signals into activating signals leading to augmented cytotoxic function in a neuroblastoma model (129).

Impaired cellular metabolism in the nutrient-poor TME is another important factor responsible for the dysregulation of NK cells (130). Glycolysis is a critical metabolic process for NK cells, especially for *ex vivo* expanded NK cells. A study by Cong et al. showed that aberrant expression of fructose bisphosphatase 1 (FBP1) in intratumoral NK cells impaired their cytotoxic function and could be a potential target to improve NK-based therapy (131). Recently, cytokine-inducible SH2-containing protein (CISH)-knockout in iPSC-derived NK cells using the CRISPR/cas9 system exhibited improved metabolic fitness as exemplified by increased basal glycolysis and mitochondrial activity, and by increased mTOR signaling which contributed to the enhanced NK cell function (132). Overall, metabolic reprograming of the TME or adoptive NK cells is a promising strategy to prevent NK cell dysfunction.

## Summary

NK cells have emerged as an attractive platform for adoptive cell immunotherapy in recent years. Key advances, including the development of efficient *ex vivo* expansion systems, the use of CIML NK cells for prolonged *in vivo* persistence and genetic manipulations strategies involving CARs have helped to catapult the field toward more effective clinical translation. However, more work needs to be done to further enhance adoptively transferred NK cells’ tumor targeting, overcome their immune suppression and exhaustion in the TME, increase their persistence in an allogeneic setting, and augment their capacity for long-term surveillance against tumor relapse. Taking advantage of recent advances in our understanding of basic NK cell biology and genetic manipulation techniques, we expect a bright future for the NK cell immunotherapy field.

## Author Contributions

MT, RR, and RS conceived of the topics to include in the review. MT and RS wrote the manuscript. MT designed the figures. RR provided critical edits to the manuscript. All authors approved of the final version.

## Funding

Funding support was provided by the Michelle D. and Douglas W. Bell Fund for Stem Cell Transplant (grant# 9618342) and the Ted and Eileen Pasquarello Research Fund (grant #9616306).

## Conflict of Interest

The authors declare that the research was conducted in the absence of any commercial or financial relationships that could be construed as a potential conflict of interest

## Publisher’s Note

All claims expressed in this article are solely those of the authors and do not necessarily represent those of their affiliated organizations, or those of the publisher, the editors and the reviewers. Any product that may be evaluated in this article, or claim that may be made by its manufacturer, is not guaranteed or endorsed by the publisher.
